# Association and Risk-Stratification Value of the Positive Remodeling Index of Middle Cerebral Artery Atherosclerotic Plaques for Perforator-Territory Infarction Based on High-Resolution Magnetic Resonance Imaging

**DOI:** 10.31083/RN53244

**Published:** 2026-07-28

**Authors:** Xiaojun Li, Yu Zhang, Huawu Zhang, Chunyang Hu, Xuebing Li

**Affiliations:** ^1^Department of Imaging, Minda Hospital of Hubei Minzu University, 445000 Enshi, Hubei, China

**Keywords:** middle cerebral artery, atherosclerotic plaque, cerebral infarction, magnetic resonance imaging, vascular remodeling

## Abstract

**Background:**

Atherosclerosis of the middle cerebral artery (MCA) is a major contributor to ischemic stroke, and infarcts confined to the perforator territory can produce considerable neurological dysfunction even when the affected volume is comparatively small. As the severity of luminal narrowing measured by conventional methods does not, on its own, account for the variety of observed infarction patterns, additional contributions from plaque remodeling and plaque vulnerability are likely. High-resolution magnetic resonance imaging (HR-MRI) permits fine-grained characterization of intracranial plaques. The present work was undertaken to examine how the positive remodeling index (PRI) of MCA atherosclerotic plaques relates to perforator-territory infarction and to provide a preliminary appraisal of the value of PRI for stratifying the risk of this infarct subtype.

**Methods:**

Patients admitted to our institution between January 2021 and December 2024 with acute ischemic stroke attributable to MCA atherosclerosis were reviewed retrospectively, yielding 283 cases. Each patient completed both conventional MRI and HR-MRI vessel wall imaging. Based on where the infarct was located, patients were separated into a perforator-territory infarction group (n = 127) and a non-perforator-territory infarction group (n = 156). The cross-sectional vessel area at the culprit lesion and at the reference segment were quantified from HR-MRI, from which the PRI was derived. Clinical and imaging variables showing an independent relationship with perforator-territory infarction were identified through univariate followed by multivariable logistic regression. The apparent discrimination of PRI was summarized using receiver operating characteristic (ROC) analysis, and the stability of the derived cutoff was examined by bootstrap resampling together with stratified 10-fold cross-validation. The added value of the PRI was judged using net reclassification improvement (NRI), integrated discrimination improvement (IDI), and decision curve analysis (DCA).

**Results:**

PRI values were greater among patients with perforator-territory infarction than among those without. In the multivariable model, perforator-territory infarction was independently linked to the PRI (odds ratio [OR] = 8.67, 95% confidence interval [CI]: 3.42−21.98, *p* < 0.001), diabetes mellitus (OR = 2.18, 95% CI: 1.21−3.92, *p* = 0.009), homocysteine (OR = 1.03, 95% CI: 1.00−1.05, *p* = 0.032), severe stenosis (OR = 2.45, 95% CI: 1.38−4.35, *p* = 0.002), marked enhancement (OR = 2.32, 95% CI: 1.28−4.21, *p* = 0.006), and intraplaque hemorrhage (OR = 1.95, 95% CI: 1.09−3.49, *p* = 0.025). Used alone, the PRI achieved moderate discrimination, with an area under the curve (AUC) of 0.728 (95% CI: 0.670−0.786). After internal validation, the bootstrap-corrected AUC was 0.716 (95% CI: 0.654−0.775) and the stratified 10-fold cross-validated AUC was 0.714 (95% CI: 0.650−0.777). A Youden-based cutoff of PRI = 1.12 was identified post hoc from the ROC curve, and bootstrap resampling located this threshold predominantly within the 1.10−1.14 interval. Incorporating the PRI into the base model raised the AUC from 0.794 to 0.832 (*p* = 0.018), accompanied by a continuous NRI of 0.318 (95% CI: 0.154−0.482) and an IDI of 0.061 (95% CI: 0.028−0.094) (both *p* < 0.001).

**Conclusions:**

Among patients with MCA atherosclerotic stroke, a higher PRI was independently related to perforator-territory infarction. Within this single-center retrospective cohort, the PRI demonstrated moderate discriminative capacity and may represent a candidate HR-MRI vessel wall imaging marker for risk stratification. Confirmation of its threshold and its place in clinical practice will require multicenter prospective investigation and validation in independent samples.

## 1. Introduction

Acute ischemic stroke remains a major cause of death and disability worldwide, and intracranial atherosclerosis accounts for an important proportion of ischemic stroke etiologies in Asian populations [1,2]. Infarcts in the deep perforator territory of the middle cerebral artery (MCA) may involve functional regions such as the basal ganglia and internal capsule; even when lesion volume is relatively small, they may cause neurological deficits or early neurological deterioration [3,4]. The degree of conventional luminal stenosis cannot fully explain differences in infarction patterns among patients, suggesting that plaque phenotype and vascular remodeling may provide additional information for risk stratification.

High-resolution magnetic resonance imaging (HR-MRI) vessel wall imaging can depict wall information beyond conventional luminal imaging, including plaque burden, enhancement, hemorrhage, and vascular remodeling, thereby providing an imaging basis for interpreting different infarction patterns under similar degrees of stenosis [5,6]. Previous studies have suggested that MCA plaque remodeling patterns, vulnerable plaque features, and white matter lesion burden may be associated with perforator-related ischemic events or stroke risk stratification; however, the independent association between the positive remodeling index (PRI) and perforator-territory infarction requires further validation [7,8]. Therefore, this study used HR-MRI vessel wall imaging to analyze the association between PRI of MCA atherosclerotic plaques and perforator-territory infarction and to preliminarily evaluate its risk-stratification value.

## 2. Materials and Methods

### 2.1 Study Population

This retrospective study drew on patients who were admitted to our institution for acute ischemic stroke during the period from January 2021 through December 2024.

To be enrolled, a patient had to satisfy all of the following: (1) being at least 18 years of age; (2) undergoing HR-MRI within 7 days of symptom onset; (3) imaging evidence of an acute infarct within the MCA territory; (4) an HR-MRI vessel wall study demonstrating an atherosclerotic plaque in the ipsilateral MCA; and (5) availability of complete clinical and imaging records. The 7-day ceiling on the onset-to-examination interval was adopted chiefly because the cohort comprised acute ischemic stroke patients, and acute-phase diffusion-weighted imaging (DWI) and fluid-attenuated inversion recovery (FLAIR) localize newly formed infarcts with reasonable consistency. Moreover, assigning the type of deep MCA infarct depends on jointly considering the acute infarct appearance and the territory supplied by the culprit artery [9,10]. Confining the imaging window in this way additionally limits how much differing treatment timing, evolving plaque enhancement, and progression of the infarct can compromise the consistency of image interpretation.

A patient was excluded if any of the following applied: (1) infarction of a non-atherosclerotic origin, for example cardioembolism, dissection of an artery, moyamoya disease, or vasculitis; (2) HR-MRI of inadequate quality, such as pronounced motion or flow artifacts or a low signal-to-noise ratio that obscured the vessel wall margins; (3) severe (≥70%) stenosis of both MCAs or acute infarction within both MCA territories; (4) serious cardiac, hepatic, or renal dysfunction, or malignancy; (5) a history of contrast-agent allergy or any contraindication to contrast administration, including severe renal impairment with an estimated glomerular filtration rate below 30 mL/min/1.73 m^2^; and (6) the lack of an acceptable reference vessel segment on either the proximal or the distal side of the plaque.

Application of these criteria left 283 patients for the final cohort. 

### 2.2 Clinical Data Collection

Baseline demographic and clinical information was retrieved for every patient, covering sex, age, and any history of hypertension, diabetes mellitus, hyperlipidemia, smoking, or prior stroke or transient ischemic attack (TIA). A patient was regarded as hypertensive if hypertension had been diagnosed previously, or if, in the absence of antihypertensive treatment, the systolic pressure reached 140 mmHg or higher and/or the diastolic pressure reached 90 mmHg or higher [11]. Diabetes mellitus was recorded when a prior diagnosis existed or when the American Diabetes Association thresholds were met, namely a fasting glucose of at least 7.0 mmol/L, glycated hemoglobin of at least 6.5%, or a random glucose of at least 11.1 mmol/L [12]. Hyperlipidemia was assigned on the basis of a previous diagnosis or, following lipid-management guidance, a total cholesterol of at least 5.2 mmol/L, low-density lipoprotein cholesterol of at least 3.4 mmol/L, or triglycerides of at least 1.7 mmol/L [13]. Patients were classified as having a smoking history if they currently smoked or had stopped less than one year earlier. A previous stroke or TIA was verified against earlier discharge diagnoses, outpatient notes, or imaging findings. The admission National Institutes of Health Stroke Scale (NIHSS) score was documented, and the onset-to-examination time was taken as the span between symptom onset and the completion of HR-MRI. Laboratory measurements obtained for analysis comprised fasting glucose, glycated hemoglobin, total cholesterol, triglycerides, low-density lipoprotein cholesterol, high-density lipoprotein cholesterol, homocysteine, and high-sensitivity C-reactive protein, all of which were assayed within 24 h of admission.

### 2.3 Imaging Examination

#### 2.3.1 Conventional Magnetic Resonance Imaging (MRI)

Imaging for all participants was carried out on a single 3.0 T MRI system (SIGNA Pioneer, GE Healthcare, Milwaukee, WI, USA) equipped with a dedicated 20-channel head coil. The conventional protocol comprised axial T1-weighted imaging (T1WI), T2-weighted imaging (T2WI), FLAIR, and DWI, with DWI acquired by an echo-planar sequence at b values of 0 and 1000 s/mm^2^. Reported as repetition time (TR) and echo time (TE), the parameters were as follows: T1WI, TR 550 ms and TE 12 ms; T2WI, TR 3500 ms and TE 100 ms; and FLAIR, TR 8500 ms and TE 150 ms, each with a 5-mm slice thickness, a 1-mm interslice gap, a 240 mm × 240 mm field of view, and a 256 × 256 matrix. For DWI, TR was 3000 ms and TE 90 ms, with the same 5-mm slice thickness and 1-mm gap, a 240 mm × 240 mm field of view, and a 128 × 128 matrix.

#### 2.3.2 High-Resolution MRI Vessel Wall Imaging

Once the affected vessel had been localized with three-dimensional time-of-flight magnetic resonance angiography (3D-TOF MRA), acquired on the same 3.0 T MRI unit, HR-MRI vessel wall imaging of the culprit MCA was performed. Coverage extended across the whole M1 segment, running from the MCA origin to the M1/M2 bifurcation, so that the diseased vessel together with its proximal and distal reference segments was captured. The acquisition consisted of axial T1WI, T2WI, and proton density-weighted imaging (PDWI), as well as contrast-enhanced T1WI obtained perpendicular to the course of the vessel. The corresponding parameters were T1WI (TR 800 ms, TE 15 ms), T2WI (TR 3200 ms, TE 50 ms), and PDWI (TR 2500 ms, TE 30 ms), with a 2-mm slice thickness, no interslice gap, a 150 mm × 150 mm field of view, a 320 × 320 matrix, and a voxel size of 0.47 mm × 0.47 mm × 2 mm. Prior to the enhanced sequence, each patient was routinely questioned about contrast-agent allergy and had renal function checked. The contrast-enhanced acquisition began 5 min after an intravenous bolus of gadopentetate dimeglumine (0.1 mmol/kg) delivered at 2 mL/s, which was chased by a 20 mL saline flush at the identical rate. Every patient successfully underwent the enhanced examination.

#### 2.3.3 Classification of Infarction Type

The pattern of infarction was judged from DWI and FLAIR images [14,15] and sorted into four categories. A perforator-territory infarct was one situated within the deep perforator (lenticulostriate) territory of the MCA, encompassing lesions of the basal ganglia and internal capsule whose largest dimension stayed below 20 mm across each of the three orthogonal DWI planes (axial, coronal, and sagittal). A watershed infarct lay in the border zone shared by the MCA and either the anterior or the posterior cerebral artery territory. A cortical/subcortical infarct affected the cortex or the subcortical white matter supplied by the MCA. A mixed infarct denoted concurrent involvement of two or more of these regions. For the analyses that followed, a mixed infarct that reached the perforator territory was counted within the perforator-territory infarction group, whereas one sparing that territory was placed in the non-perforator-territory infarction group. Two neuroradiologists, each with over five years of experience in interpreting stroke imaging and each unaware of the quantitative HR-MRI plaque measurements, independently classified infarction type from the DWI and FLAIR images. Where their assessments diverged, a third senior neuroradiologist reviewed the case and a consensus was reached.

### 2.4 HR-MRI Image Analysis and Measurement of Plaque Parameters

Two neuroradiologists skilled in neurovascular wall imaging carried out the quantitative HR-MRI plaque measurements independently (reader A had 10 years of diagnostic neuroimaging experience and 6 years specific to HR-MRI vessel wall imaging; reader B had 8 and 5 years, respectively). Ahead of measurement, a study coordinator used the clinical presentation together with DWI, 3D-TOF MRA, and HR-MRI to designate the culprit-side MCA and the candidate lesion segment, then handed the readers that vessel segment with all infarction-type labels stripped away. Throughout the quantitative work, both readers remained masked to each patient’s clinical history, laboratory values, and ultimate infarction-type assignment. Analysis was conducted with dedicated vascular software (VesselMass, version 2021; Leiden University Medical Center, Leiden, The Netherlands).

#### 2.4.1 Plaque Localization and Assessment of Stenosis Severity

A plaque was deemed the culprit when it sat in the ipsilateral M1 segment that matched the acute infarct, occupied the same vascular pathway as the DWI-delineated acute lesion, and could account for the ischemia in the downstream territory. When more than one plaque was found in the ipsilateral MCA, preference was given to the lesion that combined the tightest stenosis, the largest plaque burden, and the closest spatial agreement with the perforator orifice or its supplied region. If the point of maximal stenosis did not coincide with the slice of maximal plaque burden, two neuroradiologists arrived at a joint decision by referring to DWI, 3D-TOF MRA, and the multisequence HR-MRI dataset. Any case in which the culprit plaque remained uncertain was dropped from the final analysis. The degree of intracranial stenosis was computed with the standardized approach proposed in the Warfarin-Aspirin Symptomatic Intracranial Disease (WASID) study and was graded as mild, moderate, or severe in line with the grading conventions widely applied to symptomatic intracranial arterial stenosis [16,17].

#### 2.4.2 Definition of Reference Vessel and Measurement of Vessel Parameters

A reference segment was taken to be a comparatively normal portion of vessel lying 5−10 mm proximal and distal to the plaque margin that was free of focal plaque, abnormal enhancement, and calcified low signal and that had a maximum wall thickness under 1.0 mm. Wall thickness was gauged on sections cut perpendicular to the vessel’s long axis and was defined as the greatest perpendicular separation between the external elastic lamina and the luminal margin. It was sampled at the 12-, 3-, 6-, and 9-o’clock positions as well as at the site judged visually thickest, with the largest of these taken as that slice’s maximum wall thickness. Qualifying segments had to be present on both the proximal and distal sides; whenever an acceptable segment was missing on either side, the patient was excluded. The reference vessel area was obtained from the qualifying proximal and distal segments and averaged before being used to compute PRI [18,19].

On the slice showing the largest plaque, the external elastic lamina (EEL) and the lumen contour were traced by hand, after which the software derived the vessel area (VA), lumen area (LA), wall area (WA = VA − LA), and plaque burden (PB = WA / VA × 100%) automatically. Each reference segment was independently verified by the two masked readers, and any proximal or distal segment exhibiting diffuse thickening, abnormal enhancement, or indistinct margins was excluded from the PRI calculation.

#### 2.4.3 Calculation of Positive Remodeling Index

VA, LA, WA, PB, and the remodeling index were quantified in keeping with expert consensus guidance on intracranial arterial vessel wall MRI and with earlier reports on intracranial arterial remodeling. PRI was expressed as the vessel area at the lesion divided by the reference vessel area, that is, PRI = VA lesion / VA reference. A PRI above 1.0 denoted positive remodeling, while a PRI at or below 1.0 indicated the absence of positive remodeling or negative remodeling [20,21].

#### 2.4.4 Analysis of Plaque Characteristics

Enhancement of the plaque was assessed quantitatively on contrast-enhanced T1WI. On the slice with the largest plaque, three regions of interest (ROIs) of roughly 2 mm^2^ each were placed to record the signal intensity of the plaque (SI_plaque) and that of the adjacent ipsilateral brain parenchyma (SI_brain). The enhancement ratio was computed as (postcontrast SI_plaque − precontrast SI_plaque) / precontrast SI_plaque × 100%, and the plaque-to-brain signal intensity ratio, termed the contrast ratio (CR), was obtained as CR = postcontrast SI_plaque / postcontrast SI_brain. Enhancement was then graded from these values: no enhancement when the enhancement ratio fell below 10%; mild enhancement when the ratio reached 10% or more while CR stayed under 0.8; and marked enhancement when CR was 0.8 or above. Plaque enhancement, intraplaque hemorrhage, lipid-rich necrotic core, and calcification were each ascertained from the signal behavior across the multisequence vessel wall images, applying an interpretive scheme consistent with that adopted in investigations of the spatial relationship between MCA perforator-related infarction and plaque [22].

### 2.5 Statistical Analysis

**Fig. 1.**
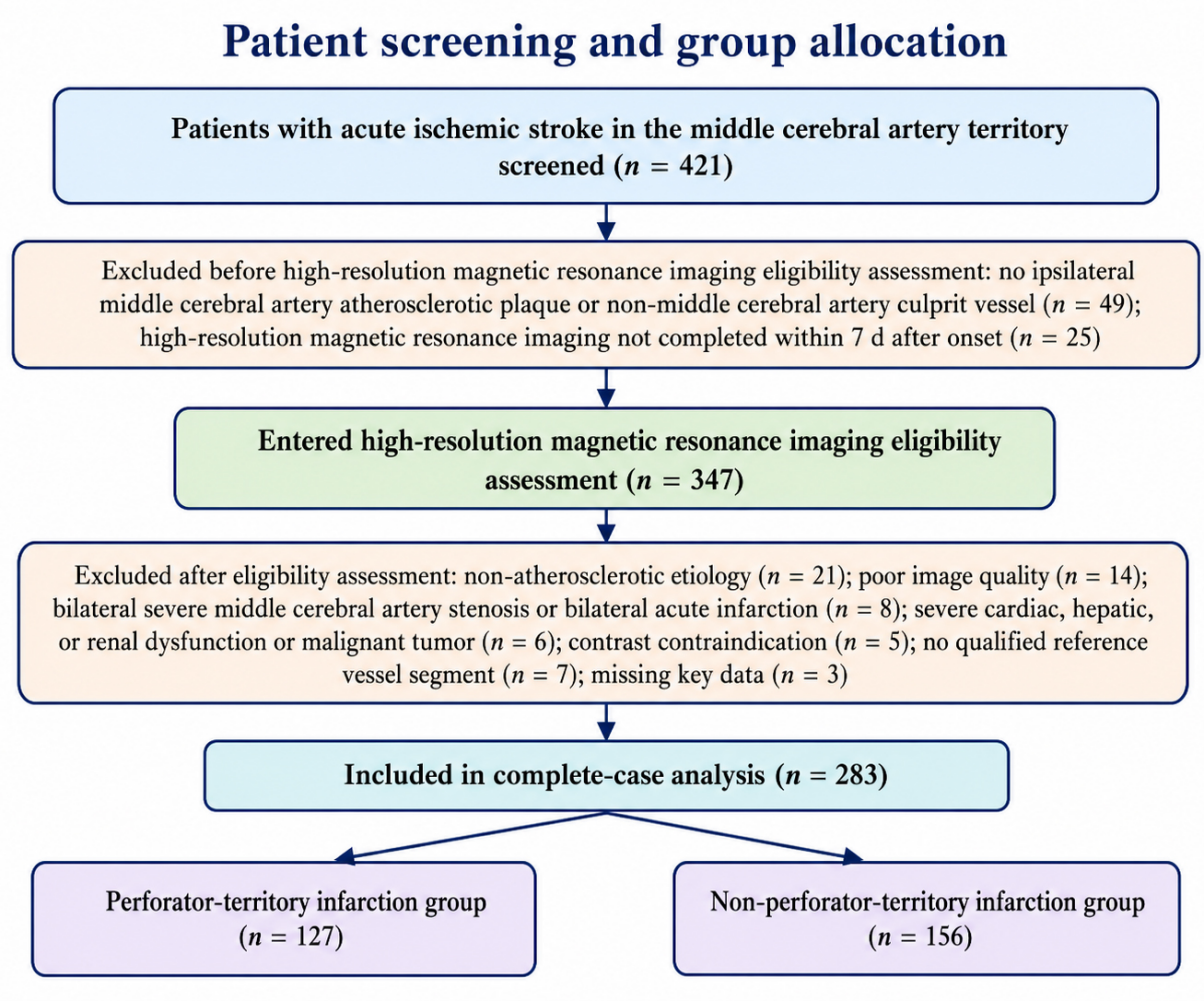
**Patient screening flowchart**.

SPSS 26.0 (IBM Corp., Armonk, NY, USA) and R 4.3.2 (R Foundation for Statistical Computing, Vienna, Austria) served as the analytical platforms. The distribution of continuous variables was checked for normality with the Shapiro-Wilk test, and variance homogeneity was examined with Levene’s test. Continuous variables that were normally distributed with homogeneous variances are reported as mean ± standard deviation and were contrasted between groups by the independent-samples *t* test, whereas those departing from normality are summarized as median with interquartile range [M(P25, P75)] and were compared by the Mann-Whitney *U* test. Categorical data appear as counts with percentages [n (%)] and were tested with the *χ^2^* test or, when appropriate, Fisher’s exact test. Missing values were addressed by a complete-case strategy. During screening, patients whose key clinical details, laboratory results, or core imaging measurements were missing and could not be confirmed from the electronic medical record were removed at the outset. Because the 283 patients ultimately analyzed had complete data for every principal modeling variable—outcome, PRI, stenosis grade, plaque enhancement, intraplaque hemorrhage, diabetes mellitus, and homocysteine—no multiple imputation was undertaken. The screening sequence and the way missing variables were distributed are shown in the patient flow diagram and an accompanying table (Fig. 1; **Supplementary Table 1**).

Factors potentially related to perforator-territory infarction were first explored by univariable logistic regression. Those reaching *p* < 0.10 in that step, supplemented by age, sex, and stenosis grade, were carried forward into the multivariable logistic model. Collinearity was checked before model entry: when two variables were strongly correlated or when the variance inflation factor (VIF) pointed to instability, the one offering greater clinical interpretability, more reproducible measurement, or a steadier link with the outcome was kept, and a discarded collinear variable was never co-entered with its counterpart. Model building proceeded by stepwise backward elimination, and its fit was appraised with the Hosmer-Lemeshow test.

ROC curves were generated and the corresponding AUCs reported with 95% confidence intervals (CIs). Within the present dataset, the best PRI cutoff was fixed post hoc as the point maximizing the Youden index on the ROC curve; this value served only for exploratory stratification and presentation and was not a clinical threshold specified in advance of the study. AUCs were compared by the DeLong test. The stability of both the AUC and the cutoff was probed with 1000 bootstrap resamples, and the robustness of PRI alone as a discriminator was checked by stratified 10-fold cross-validation. The incremental contribution of PRI beyond the base clinical-imaging model was quantified through the net reclassification improvement (NRI), the integrated discrimination improvement (IDI), and decision curve analysis (DCA). The overall analysis plan, the variable-selection approach, the treatment of collinearity, the internal validation, the sensitivity analyses, and the NRI/IDI/DCA procedures were each independently scrutinized by a statistician versed in clinical research methodology. Every test was two-sided, and statistical significance was set at *p* < 0.05. This retrospective observational study was reported in accordance with the STROBE statement, and the completed STROBE checklist is provided as **Supplementary Material-STROBE_Checklist**.

## 3. Results

### 3.1 Patient Screening and Baseline Characteristics

During the study period, 421 patients with acute ischemic stroke in the MCA territory were screened. After excluding 49 patients with no ipsilateral MCA atherosclerotic plaque or with a non-MCA culprit vessel and 25 patients who did not complete HR-MRI within 7 d after onset, 347 patients entered the HR-MRI eligibility assessment. Additional exclusions were non-atherosclerotic etiology (n = 21), poor image quality (n = 14), bilateral severe MCA stenosis or bilateral acute infarction (n = 8), severe cardiac, hepatic, or renal dysfunction or malignant tumor (n = 6), contrast contraindication (n = 5), lack of a qualified reference vessel segment (n = 7), and missing key data (n = 3). Finally, 283 complete cases were included in the analysis (Fig. 1; **Supplementary Table 1**).

A total of 283 patients were included, comprising 127 in the perforator-territory infarction group and 156 in the non-perforator-territory infarction group. The two groups differed significantly in hypertension, diabetes mellitus, NIHSS score, fasting blood glucose, glycated hemoglobin, triglycerides, homocysteine, and high-sensitivity C-reactive protein. Overall, hypertension, diabetes mellitus, and metabolic-inflammatory indicators were higher in the perforator-territory infarction group, whereas NIHSS scores were lower than in the non-perforator-territory infarction group. Age, sex, hyperlipidemia, smoking history, previous stroke/TIA, time from onset to HR-MRI examination, total cholesterol, low-density lipoprotein cholesterol, and high-density lipoprotein cholesterol did not differ significantly between groups (all *p* > 0.05) (Table 1).

**Table 1.** **Baseline clinical characteristics of the patients**.

Clinical characteristic	Overall (n = 283)	Perforator group (n = 127)	Non-perforator group (n = 156)	Statistic	*p* value
Age, years	61.5 ± 10.8	62.8 ± 10.3	60.4 ± 11.1	*t* = 1.838	0.067
Male [n (%)]	186 (65.7)	88 (69.3)	98 (62.8)	*χ^2^* = 1.323	0.250
Hypertension [n (%)]	215 (76.0)	106 (83.5)	109 (69.9)	*χ^2^* = 7.120	0.008
Diabetes mellitus [n (%)]	128 (45.2)	71 (55.9)	57 (36.5)	*χ^2^* = 10.640	0.001
Hyperlipidemia [n (%)]	167 (59.0)	78 (61.4)	89 (57.1)	*χ^2^* = 0.550	0.458
Smoking history [n (%)]	142 (50.2)	68 (53.5)	74 (47.4)	*χ^2^* = 1.078	0.298
Previous stroke/transient ischemic attack [n (%)]	89 (31.4)	45 (35.4)	44 (28.2)	*χ^2^* = 1.756	0.185
National institutes of health stroke scale score [M(P25, P75)]	5 (3, 8)	4 (2, 6)	6 (4, 9)	*Z* = –4.125	<0.001
Time from onset to high-resolution magnetic resonance imaging, d [M(P25, P75)]	3 (2, 5)	3 (2, 4)	3 (2, 5)	*Z* = –1.012	0.312
Fasting blood glucose (mmol/L)	6.2 ± 2.1	6.6 ± 2.3	5.9 ± 1.9	*t* = 2.759	0.006
Glycated hemoglobin (%)	6.4 ± 1.3	6.7 ± 1.5	6.2 ± 1.2	*t* = 3.008	0.003
Total cholesterol (mmol/L)	4.8 ± 1.2	4.9 ± 1.3	4.7 ± 1.1	*t* = 1.327	0.185
Triglycerides (mmol/L) [M(P25, P75)]	1.6 (1.2, 2.3)	1.7 (1.3, 2.5)	1.5 (1.1, 2.1)	*Z* = –2.034	0.042
Low-density lipoprotein cholesterol (mmol/L)	2.9 ± 0.9	3.0 ± 0.9	2.8 ± 0.8	*t* = 1.745	0.082
High-density lipoprotein cholesterol (mmol/L)	1.2 ± 0.3	1.1 ± 0.3	1.2 ± 0.3	*t* = –1.530	0.127
Homocysteine (μmol/L) [M(P25, P75)]	14.2 (10.5, 19.8)	16.1 (11.8, 21.5)	13.1 (9.8, 18.2)	*Z* = –2.652	0.008
High-sensitivity C-reactive protein (mg/L) [M(P25, P75)]	3.8 (1.5, 8.2)	4.5 (2.1, 9.6)	3.2 (1.2, 6.8)	*Z* = –2.428	0.015

Note: The perforator group refers to the perforator-territory infarction group, and the non-perforator group refers to the non-perforator-territory infarction group. n, number of cases; %, percentage; M(P25, P75), median (25th percentile, 75th percentile); *t*, independent-samples *t*-test statistic; *χ^2^*, chi-square test statistic; *Z*, standardized statistic of the Mann-Whitney *U* test; *p* value, two-sided probability of significance.

### 3.2 HR-MRI Vessel Wall Imaging Characteristics

The perforator-territory infarction group had a higher proportion of severe stenosis and higher VA, WA, PB, PRI, proportion of PRI > 1.0, plaque enhancement ratio, plaque/brain signal intensity ratio, marked enhancement, intraplaque hemorrhage, and lipid-rich necrotic core than the non-perforator-territory infarction group, whereas LA was lower in the perforator-territory infarction group (all *p* < 0.05). No significant differences were observed in reference vessel area or calcification proportion between the two groups (both *p* > 0.05) (Table 2).

**Table 2.** **Comparison of high-resolution magnetic resonance imaging vessel wall parameters and plaque characteristics between groups**.

Imaging parameter		Perforator group (n = 127)	Non–perforator group (n = 156)	Statistic	*p* value
Stenosis grade [n (%)]				Pearson *χ^2^* = 8.651	0.013
	Mild (<50%)	18 (14.2)	39 (25.0)		
	Moderate (50%−69%)	30 (23.6)	46 (29.5)		
	Severe (70%−99%)	79 (62.2)	71 (45.5)		
Reference vessel area (mm^2^)		12.3 ± 2.8	12.1 ± 2.6	*t* = 0.611	0.542
Vessel area (mm^2^)		14.6 ± 3.5	12.8 ± 3.1	*t* = 4.578	<0.001
Lumen area (mm^2^)		4.8 ± 2.1	5.6 ± 2.3	*t* = –3.011	0.003
Wall area (mm^2^)		9.8 ± 2.4	7.2 ± 2.1	*t* = 9.652	<0.001
Plaque burden (%)		67.1 ± 12.3	56.3 ± 13.8	*t* = 6.875	<0.001
Positive remodeling index (PRI)		1.18 ± 0.21	1.06 ± 0.18	*t* = 5.164	<0.001
PRI >1.0 [n (%)]		100 (78.7)	86 (55.1)	Pearson *χ^2^* = 17.326	<0.001
Plaque enhancement ratio (%)		45.8 ± 18.6	32.4 ± 17.2	*t* = 6.221	<0.001
Plaque/brain signal intensity ratio (contrast ratio [CR])		0.89 ± 0.25	0.72 ± 0.23	*t* = 5.926	<0.001
Plaque enhancement grade [n (%)]				Pearson *χ^2^* = 21.430	<0.001
	No enhancement	12 (9.4)	35 (22.4)		
	Mild enhancement	38 (29.9)	68 (43.6)		
	Marked enhancement	77 (60.6)	53 (34.0)		
Intraplaque hemorrhage [n (%)]		68 (53.5)	52 (33.3)	Pearson *χ^2^* = 11.708	0.001
Lipid-rich necrotic core [n (%)]		91 (71.7)	87 (55.8)	Pearson *χ^2^* = 7.569	0.006
Calcification [n (%)]		42 (33.1)	48 (30.8)	Pearson *χ^2^* = 0.171	0.679

Note: CR, contrast ratio; n, number of cases; %, percentage; *t*, independent-samples *t*-test statistic; Pearson *χ^2^*, Pearson chi-square test statistic; *p* value, two-sided probability of significance. Stenosis grade was categorized as mild (<50%), moderate (50%−69%), and severe (70%−99%) according to stenosis rate.

A random sample of 60 patients was selected for reproducibility analysis. The interobserver intraclass correlation coefficients (ICCs) for lesion VA, reference vessel area, LA, WA, PB, PRI, plaque enhancement ratio, and CR were 0.91, 0.89, 0.90, 0.90, 0.90, 0.88, 0.87, and 0.86, respectively; the corresponding intraobserver ICCs were 0.94, 0.92, 0.93, 0.93, 0.93, 0.91, 0.90, and 0.89. The interobserver Kappa coefficients for marked enhancement, intraplaque hemorrhage, lipid-rich necrotic core, and calcification ranged from 0.78 to 0.86, and the intraobserver Kappa coefficients ranged from 0.82 to 0.90, indicating good reproducibility of quantitative and qualitative HR-MRI evaluations (**Supplementary Table 2**).

### 3.3 Multivariable Logistic Regression Analysis

Variables with *p* < 0.10 in univariable analysis, together with age, sex, and stenosis severity, were entered into the multivariable analysis. Collinearity diagnostics showed high collinearity between fasting blood glucose and glycated hemoglobin, between VA and WA, between WA and PB, between plaque enhancement ratio and plaque/brain signal intensity ratio, and between plaque/brain signal intensity ratio and marked enhancement. Variables with stronger clinical interpretability and more stable associations with the outcome were ultimately retained. After adjustment, PRI, diabetes mellitus, homocysteine, severe stenosis, marked enhancement, and intraplaque hemorrhage were independently associated with perforator-territory infarction (all *p* < 0.05) (Table 3). Univariable logistic regression analysis is shown in **Supplementary Table 3**.

**Table 3.** **Multivariable logistic regression analysis of perforator-territory infarction**.

Variable	*β*	SE	OR	95% CI	*p* value
Diabetes mellitus	0.779	0.298	2.18	1.21–3.92	0.009
Homocysteine	0.026	0.012	1.03	1.00–1.05	0.032
Severe stenosis	0.895	0.290	2.45	1.38–4.35	0.002
Positive remodeling index (PRI)	2.160	0.473	8.67	3.42–21.98	<0.001
Marked enhancement	0.843	0.307	2.32	1.28–4.21	0.006
Intraplaque hemorrhage	0.668	0.298	1.95	1.09–3.49	0.025

Note: Logistic regression refers to binary logistic regression. *β*, regression coefficient; SE, standard error; OR, odds ratio; CI, confidence interval; *p* value, two-sided probability of significance.

### 3.4 Discriminative Ability, Internal Validation, and Incremental Value

**Fig. 2.**
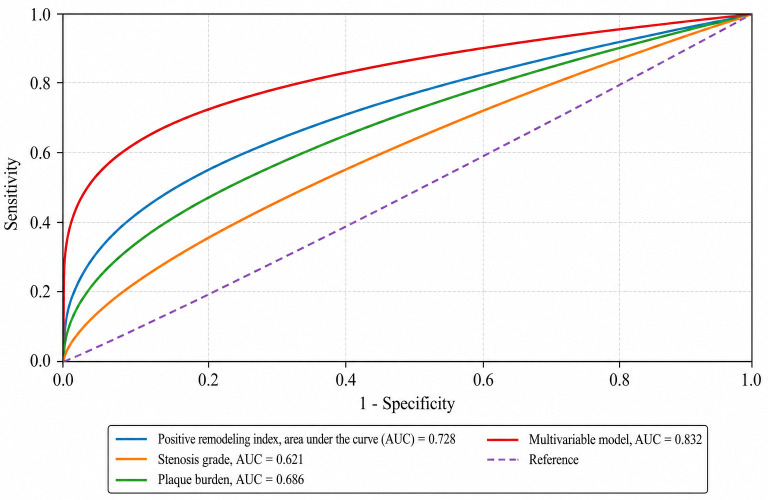
**ROC curves of the PRI, stenosis severity, plaque burden, and multivariable model for perforator-territory infarction**.

**Fig. 3.**
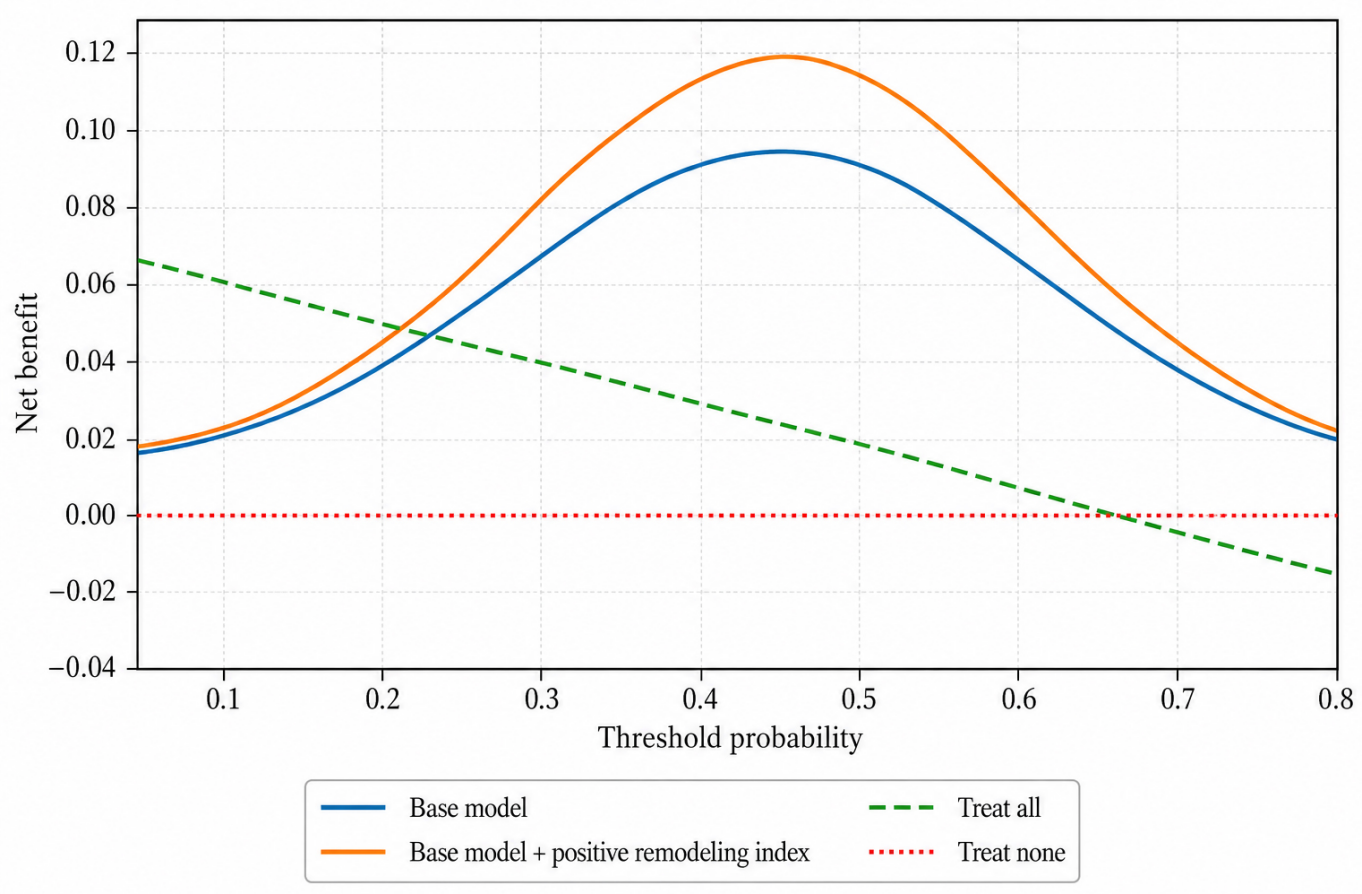
**DCA of the base model and base model + PRI**.

ROC curve analysis showed that PRI had moderate discriminative ability for perforator-territory infarction. Its discriminative ability was higher than that of stenosis severity and plaque burden but lower than that of the multivariable model (all *p* < 0.05) (Fig. 2; Table 4). Bootstrap resampling and stratified 10-fold cross-validation suggested mild optimism in the discriminative performance of PRI as a single indicator but overall stability. PRI = 1.12 was a post hoc ROC-derived Youden cutoff, and bootstrap resampling showed that this cutoff was mainly distributed between 1.10 and 1.14. The AUC of the base model was 0.794 (95% CI: 0.741−0.847), and the AUC of the base model + PRI was 0.832 (95% CI: 0.783−0.881); the DeLong test showed a statistically significant difference between the two models (*p* = 0.018). The continuous NRI was 0.318 (95% CI: 0.154−0.482), and the IDI was 0.061 (95% CI: 0.028−0.094) (both *p* < 0.001). DCA showed that within a threshold probability range of 0.20−0.70, the base model + PRI generally had a higher net benefit than the base model. This result reflects only the potential clinical incremental value within the sample of the present study (Fig. 3; Table 4).

**Table 4.** **Discriminative ability, internal validation, and incremental value of positive remodeling index (PRI)**.

Indicator	Value	Statistical method	*p* value/Remark
PRI apparent AUC	0.728 (95% CI: 0.670–0.786)	ROC	Not applicable
PRI bootstrap-corrected AUC	0.716 (95% CI: 0.654–0.775)	1000 bootstrap resamples	Not applicable
PRI 10-fold cross-validated AUC	0.714 (95% CI: 0.650–0.777)	Stratified 10-fold cross-validation	Not applicable
PRI cutoff stability	Median 1.12 (IQR: 1.10–1.14); 72.6% located between 1.10 and 1.14	1000 bootstrap resamples + Youden index	Not applicable
Base model AUC	0.794 (95% CI: 0.741–0.847)	ROC	Not applicable
Base model + PRI AUC	0.832 (95% CI: 0.783–0.881)	ROC/DeLong	0.018
Continuous NRI	0.318 (95% CI: 0.154–0.482)	Category-free NRI	<0.001
IDI	0.061 (95% CI: 0.028–0.094)	IDI	<0.001
DCA	Within the threshold probability range of 0.20–0.70, the model + PRI showed generally higher net benefit	Decision curve analysis	Not applicable

Note: AUC, area under the curve; ROC, receiver operating characteristic; IQR, interquartile range; NRI, net reclassification improvement; IDI, integrated discrimination improvement; DCA, decision curve analysis; DeLong, nonparametric test for comparing differences in AUC; *p* value, two-sided probability of significance.

### 3.5 PRI Threshold Stratification and Sensitivity Analyses

Threshold-stratified analysis based on PRI = 1.12 showed that the proportion of perforator-territory infarction was higher in the high-PRI group than in the low-PRI group, and this trend was consistent in both severe stenosis and non-severe stenosis subgroups (all *p* < 0.001) (**Supplementary Table 4**). Sensitivity analyses showed that when glycated hemoglobin was substituted for diabetes mellitus, CR was substituted for marked enhancement, PB was included instead of area-related variables, or the model of clinical variables + stenosis severity + PRI was used, the direction and statistical significance of the independent association between PRI and perforator-territory infarction remained consistent (**Supplementary Table 5**).

## 4. Discussion

This study used HR-MRI vessel wall imaging to systematically analyze plaque characteristics in 283 patients with MCA atherosclerotic stroke. The results showed that PRI was higher in patients with perforator-territory infarction than in those with non-perforator-territory infarction. After adjustment for diabetes mellitus, homocysteine, stenosis severity, plaque enhancement, and intraplaque hemorrhage, PRI remained independently associated with perforator-territory infarction. PRI as a single indicator showed moderate discriminative ability (AUC = 0.728), and the multivariable model showed good discriminative ability (AUC = 0.832). It should be emphasized that this study was a single-center retrospective analysis; the results support statistical association and risk-stratification value but cannot prove a causal relationship between PRI and perforator-territory infarction [23].

The epidemiology, pathophysiology, and imaging manifestations of intracranial atherosclerosis vary across populations; therefore, the applicability of single-center results to different regions and patient populations should be interpreted cautiously [24]. A meta-analysis of vessel wall imaging showed that features such as plaque enhancement, remodeling, and hemorrhage are associated with the risk of first-ever or recurrent ischemic events, supporting the incorporation of vessel wall information into risk-stratification frameworks [25]. In symptomatic intracranial atherosclerosis, HR-MRI features such as plaque enhancement, plaque burden, and remodeling are associated with recurrent ischemic events, suggesting that stenosis severity alone may be insufficient for comprehensive risk assessment [26].

This study observed an independent association between elevated PRI and perforator-territory infarction, while the perforator-territory infarction group also had higher proportions of intraplaque hemorrhage, lipid-rich necrotic core, and marked enhancement. These findings support the statistical association that positively remodeled plaques may participate in perforator-territory infarction risk stratification together with plaque vulnerability. However, this study did not directly visualize compression, distortion, or plaque coverage of perforator artery orifices or *in situ* thrombosis. Therefore, the explanation that positive remodeling affects perforator artery orifices should be considered a mechanistic hypothesis based on previous pathological and imaging studies rather than a conclusion directly demonstrated by this study.

Diabetes mellitus may influence the progression of intracranial atherosclerosis through endothelial injury, inflammatory responses, and changes in plaque phenotype, but this study only supports a statistical association between diabetes mellitus and perforator-territory infarction [27]. Elevated homocysteine is associated with ischemic stroke risk and may involve oxidative stress and vascular endothelial dysfunction; however, its effect size in this study was small and should be interpreted cautiously in the clinical context [28]. Longitudinal vessel wall imaging studies have suggested that vessel wall signal and stenosis status of symptomatic intracranial lesions may change over time, indicating that a single cross-sectional measurement cannot fully reflect the dynamic plaque process [29].

Four-dimensional flow magnetic resonance imaging (4D flow MRI) studies suggest hemodynamic differences across anatomical locations within the MCA, which may be related to local plaque formation and distribution [30]. Computational fluid dynamics provides a methodological basis for evaluating local wall shear stress, pressure gradients, and flow disturbance and may help further explain the potential links among plaque remodeling and downstream ischemic events [31]. Early computational fluid dynamics studies have shown that hemodynamic parameters of symptomatic intracranial arterial stenosis may be associated with recurrence risk, providing a basis for future integrated modeling with HR-MRI [32].

Emerging vessel wall MRI markers such as edge-type hyperintensity may reflect plaque activity and may be combined with PRI to explore risk stratification for perforator-related ischemia [33]. Interactions may exist between intracranial atherosclerosis and cerebral small vessel disease imaging manifestations such as white matter hyperintensities, and these factors may influence the occurrence and imaging appearance of perforator-territory ischemia [34]. Longitudinal MRI studies of intracranial plaque evolution and vessel wall remodeling have shown that plaque morphology and remodeling patterns are dynamic, suggesting that the risk-stratification value of PRI requires further confirmation in repeated-measurement studies [35].

Follow-up HR-MRI studies suggest that plaque evolution can predict subsequent ischemic events, providing a research basis for dynamic monitoring of MCA plaque phenotype changes [36]. Studies of combined head-and-neck high-resolution vessel wall imaging (HR-VWI) suggest that intracranial and carotid plaque phenotypes may jointly influence stroke recurrence risk, and future studies may further assess the effect of multivessel-bed plaque burden on PRI-based risk stratification [37]. HR-MRI-based recurrence risk models suggest that combining quantitative plaque features with clinical variables may improve risk discrimination, but model generalizability remains dependent on scanning protocols and validation samples [38].

This study has several limitations. First, it was a single-center retrospective analysis, which may be subject to selection bias and cannot support causal inference. Second, PRI = 1.12 was derived from this study dataset and cannot be directly used as a broad clinical decision-making threshold. Third, HR-MRI cannot directly visualize all perforator artery orifices or microthrombus formation; therefore, mechanistic explanations should remain cautious. Fourth, all scans were obtained using the same scanner and protocol, and applicability across different scanners, sequence parameters, and populations requires validation.

Future studies may adopt multicenter prospective designs, include independent validation samples, and combine longitudinal HR-MRI, perforator artery visualization, 4D flow MRI, and computational fluid dynamics to evaluate the temporal relationships among plaque remodeling, local hemodynamic changes, and perforator-territory infarction.

## 5. Conclusions

Elevated PRI of MCA atherosclerotic plaques was independently associated with perforator-territory infarction. When combined with plaque enhancement, intraplaque hemorrhage, and traditional risk factors, PRI improved risk discrimination within the present study sample. HR-MRI vessel wall imaging can provide noninvasive imaging information for assessing MCA plaque remodeling patterns; however, the cutoff value PRI = 1.12 was derived from this study dataset and requires external validation before broad clinical decision-making use.

## Data Availability

The datasets generated and analyzed during the current study are available from the corresponding author on reasonable request.
