# Supplementary material for: Association and Risk-Stratification Value of the Positive Remodeling Index of Middle Cerebral Artery Atherosclerotic Plaques for Perforator-Territory Infarction Based on High-Resolution Magnetic Resonance Imaging
Source: Rev Neurol. 2026 Jul 28;81(7):53244. doi: 10.31083/RN53244 (PMC13421104; doi:10.31083/RN53244)
Supplement: Supplementary file 1 [file 1576-6578-81-7-53244-s1.zip › Supplementary Tables.docx]

**Supplementary Materials**

**Supplementary Table 1. Distribution of missing data and reasons for exclusion during screening**

| **Variable/reason excluded at screening stage** | **No. of cases excluded** | **Description** |
| --- | --- | --- |
| No ipsilateral MCA atherosclerotic plaque or non-MCA culprit vessel | 49 | Excluded before HR-MRI eligibility assessment |
| HR-MRI not completed within 7 d after onset | 25 | Excluded before HR-MRI eligibility assessment |
| Non-atherosclerotic etiology | 21 | Cardioembolism, dissection, vasculitis, etc. |
| Poor image quality | 14 | Motion artifacts, flow artifacts, or unclear boundaries |
| Bilateral severe MCA stenosis or bilateral acute infarction | 8 | A unilateral culprit plaque could not be reliably determined |
| Severe cardiac, hepatic, or renal dysfunction or malignant tumor | 6 | Met exclusion criteria |
| Contrast contraindication | 5 | Contrast-enhanced evaluation could not be completed |
| Lack of a qualified reference vessel segment | 7 | Proximal or distal reference segment did not meet criteria |
| Missing key data | 3 | One case missing glycated hemoglobin, one case missing high-sensitivity C-reactive protein, and one case with unverifiable smoking history/previous stroke or TIA information |
| Final complete-case analysis included | 283 | No missing data in the main modeling variables |

Note: MCA, middle cerebral artery; HR-MRI, high-resolution magnetic resonance imaging; HbA1c, glycated hemoglobin; hsCRP, high-sensitivity C-reactive protein; TIA, transient ischemic attack. The number of exclusions refers to cases excluded for the corresponding reason during the screening stage.

**Supplementary Table 2. Imaging measurement agreement**

| **Measure** | **Interobserver agreement** | **Intraobserver agreement** | **Statistical method** |
| --- | --- | --- | --- |
| Lesion vessel area (VA) | 0.91 (0.86-0.95) | 0.94 (0.90-0.97) | Two-way random-effects ICC |
| Reference vessel area | 0.89 (0.83-0.94) | 0.92 (0.87-0.96) | Two-way random-effects ICC |
| Lumen area (LA) | 0.90 (0.84-0.94) | 0.93 (0.88-0.96) | Two-way random-effects ICC |
| Wall area (WA) | 0.90 (0.85-0.94) | 0.93 (0.89-0.96) | Two-way random-effects ICC |
| Plaque burden (PB) | 0.90 (0.85-0.94) | 0.93 (0.89-0.96) | Two-way random-effects ICC |
| Positive remodeling index (PRI) | 0.88 (0.82-0.93) | 0.91 (0.86-0.95) | Two-way random-effects ICC |
| Plaque enhancement ratio | 0.87 (0.80-0.92) | 0.90 (0.84-0.94) | Two-way random-effects ICC |
| Contrast ratio (CR) | 0.86 (0.79-0.92) | 0.89 (0.83-0.94) | Two-way random-effects ICC |
| Marked enhancement | *κ* = 0.82 (0.74-0.90) | *κ* = 0.86 (0.78-0.94) | Kappa |
| Intraplaque hemorrhage | *κ* = 0.84 (0.76-0.92) | *κ* = 0.89 (0.81-0.97) | Kappa |
| Lipid-rich necrotic core | *κ* = 0.78 (0.68-0.88) | *κ* = 0.82 (0.72-0.92) | Kappa |
| Calcification | *κ* = 0.86 (0.78-0.94) | *κ* = 0.90 (0.82-0.98) | Kappa |

Note: VA, vessel area; LA, lumen area; WA, wall area; PB, plaque burden; PRI, positive remodeling index; CR, contrast ratio; ICC, intraclass correlation coefficient calculated using a two-way random-effects model; *κ*, kappa coefficient; Kappa was used to evaluate agreement for categorical variables; values in parentheses are 95% CIs.

**Supplementary Table 3. Univariable logistic regression analysis of perforator-territory infarction**

| **Variable** | ***β*** | **SE** | **OR** | **95% CI** | **Wald *χ²*** | ***P* value** |
| --- | --- | --- | --- | --- | --- | --- |
| Age | 0.022 | 0.012 | 1.02 | 1.00-1.04 | 3.34 | 0.068 |
| Male | 0.293 | 0.253 | 1.34 | 0.82-2.19 | 1.339 | 0.251 |
| Hypertension | 0.775 | 0.294 | 2.17 | 1.22-3.86 | 6.945 | 0.008 |
| Diabetes mellitus | 0.788 | 0.242 | 2.20 | 1.37-3.53 | 10.604 | 0.001 |
| Hyperlipidemia | 0.177 | 0.239 | 1.19 | 0.75-1.90 | 0.548 | 0.459 |
| Smoking history | 0.246 | 0.235 | 1.28 | 0.81-2.02 | 1.096 | 0.298 |
| Previous stroke/TIA | 0.329 | 0.250 | 1.39 | 0.85-2.27 | 1.733 | 0.186 |
| NIHSS score | -0.117 | 0.032 | 0.89 | 0.84-0.95 | 13.387 | <0.001 |
| Fasting blood glucose | 0.158 | 0.058 | 1.17 | 1.05-1.31 | 7.454 | 0.006 |
| Glycated hemoglobin | 0.293 | 0.101 | 1.34 | 1.10-1.63 | 8.414 | 0.004 |
| Total cholesterol | 0.131 | 0.100 | 1.14 | 0.94-1.39 | 1.722 | 0.186 |
| Triglycerides | 0.247 | 0.123 | 1.28 | 1.01-1.62 | 4.043 | 0.044 |
| Low-density lipoprotein cholesterol | 0.223 | 0.129 | 1.25 | 0.97-1.61 | 2.989 | 0.083 |
| High-density lipoprotein cholesterol | -0.494 | 0.331 | 0.61 | 0.32-1.17 | 2.228 | 0.129 |
| Homocysteine | 0.027 | 0.010 | 1.03 | 1.01-1.05 | 6.832 | 0.009 |
| High-sensitivity C-reactive protein | 0.047 | 0.020 | 1.05 | 1.01-1.09 | 5.642 | 0.017 |
| Severe stenosis (70%-99%) | 0.683 | 0.221 | 1.98 | 1.28-3.06 | 9.558 | 0.002 |
| Vessel area | 0.166 | 0.041 | 1.18 | 1.09-1.28 | 16.422 | <0.001 |
| Lumen area | -0.151 | 0.057 | 0.86 | 0.77-0.96 | 7.009 | 0.008 |
| Wall area | 0.501 | 0.088 | 1.65 | 1.39-1.96 | 32.416 | <0.001 |
| Plaque burden | 0.062 | 0.012 | 1.06 | 1.04-1.09 | 27.562 | <0.001 |
| Positive remodeling index (PRI) | 2.513 | 0.434 | 12.35 | 5.28-28.91 | 33.522 | <0.001 |
| Plaque enhancement ratio | 0.039 | 0.009 | 1.04 | 1.02-1.06 | 18.754 | <0.001 |
| Plaque/brain signal intensity ratio | 1.522 | 0.386 | 4.58 | 2.15-9.76 | 15.558 | <0.001 |
| Marked enhancement | 1.091 | 0.246 | 2.98 | 1.84-4.83 | 19.677 | <0.001 |
| Intraplaque hemorrhage | 0.834 | 0.244 | 2.30 | 1.42-3.72 | 11.698 | 0.001 |
| Lipid-rich necrotic core | 0.703 | 0.251 | 2.02 | 1.24-3.29 | 7.850 | 0.005 |
| Calcification | 0.104 | 0.249 | 1.11 | 0.68-1.81 | 0.175 | 0.624 |

Note: Logistic regression refers to binary logistic regression. *β*, regression coefficient; SE, standard error; OR, odds ratio; CI, confidence interval; Wald *χ²*, Wald test chi-square statistic; *P* value, two-sided probability of significance; TIA, transient ischemic attack; NIHSS, National Institutes of Health Stroke Scale; PRI, positive remodeling index.

**Supplementary Table 4. Threshold-stratified analysis of positive remodeling index (PRI)**

| **Stratum** | **Sample size** | **Event distribution** | ***χ²*** | ***P* value** |
| --- | --- | --- | --- | --- |
| All patients | Low PRI n = 142; high PRI n = 141 | Perforator 36 vs 91; non-perforator 106 vs 50 | *χ²* = 43.918 | <0.001 |
| Patients with severe stenosis | Low PRI n = 58; high PRI n = 92 | Perforator 20 vs 59; non-perforator 38 vs 33 | *χ²* = 12.543 | <0.001 |
| Patients without severe stenosis | Low PRI n = 84; high PRI n = 49 | Perforator 16 vs 32; non-perforator 68 vs 17 | *χ²* = 28.711 | <0.001 |

Note: PRI, positive remodeling index; n, number of cases; *χ²*, Pearson chi-square test statistic; *P* value, two-sided probability of significance. The low-PRI group was defined as PRI < 1.12, and the high-PRI group was defined as PRI >= 1.12.

**Supplementary Table 5. Sensitivity analyses**

| **Sensitivity model** | **Key result** | **Model variables/replacement strategy** | **Interpretation** |
| --- | --- | --- | --- |
| Main final model | PRI OR = 8.67 (95% CI: 3.42-21.98), *P* < 0.001; model AUC = 0.832 | Age, sex, diabetes mellitus, homocysteine, severe stenosis, PRI, marked enhancement, and intraplaque hemorrhage | PRI remained independently associated with perforator-territory infarction |
| Glycated hemoglobin substituted for diabetes mellitus | PRI OR = 8.41 (95% CI: 3.30-21.43), *P* < 0.001; model AUC = 0.829 | Glycated hemoglobin substituted for diabetes mellitus; other candidate variables were consistent with the main analysis | After replacement of the glycometabolic variable, the direction and statistical significance of the PRI association remained consistent |
| CR substituted for marked enhancement | PRI OR = 8.24 (95% CI: 3.28-20.72), *P* < 0.001; model AUC = 0.827 | Plaque/brain signal intensity ratio (CR) substituted for marked enhancement; other candidate variables were consistent with the main analysis | After replacement of the enhancement indicator, the direction and statistical significance of the PRI association remained consistent |
| Plaque burden included without vessel area/wall area | PRI OR = 7.96 (95% CI: 3.14-20.18), *P* < 0.001; model AUC = 0.835 | Plaque burden was included, avoiding simultaneous entry of vessel area, wall area, and plaque burden into the model | After replacement of area-related variables, the direction and statistical significance of the PRI association remained consistent |
| Clinical variables + stenosis severity + PRI model | PRI OR = 8.92 (95% CI: 3.61-22.05), *P* < 0.001; model AUC = 0.806 | Clinical variables, stenosis severity, and PRI were included; enhancement and plaque-component variables were not included | PRI remained independently associated even without enhancement and plaque-component variables |

Note: PRI, positive remodeling index; OR, odds ratio; CI, confidence interval; AUC, area under the curve; CR, contrast ratio. Glycated hemoglobin was used to substitute for the diabetes mellitus variable. *P* value, two-sided probability of significance.
